# Development and validation of a new method for locating patella sensory nerves for the treatment of inferior and superior knee pain

**DOI:** 10.1186/s40634-015-0032-2

**Published:** 2015-08-19

**Authors:** Emily Hu, Jessica Preciado, Vinod Dasa, Jason Mussell

**Affiliations:** myoscience, Inc., 46400 Fremont Blvd, Fremont, CA 94538 USA; Department of Orthopedics, Louisiana State University, 1542 Tulane Avenue, Box T6-7, New Orleans, LA 70112 USA; Department of Cell Biology and Anatomy, Louisiana State University, 1901 Perdido Street, Room 6123A, New Orleans, LA 70112 USA

**Keywords:** Anterior knee pain, Infrapatellar nerves, Saphenous nerve, Femoral cutaneous nerves, Cryoneurolysis

## Abstract

**Background:**

Radiofrequency ablation and percutaneous cryoneurolysis to relieve knee pain requires treating large areas to ensure coverage due to high variability in the sensory innervation of the knee and limitations of current methods for defining treatment targets. This study sought to define and validate a new treatment approach targeting the major sensory nerves of the superior patella and expand upon previous work to define a more efficient treatment approach targeting the sensory nerves of the inferior patella.

**Methods:**

Transcutaneous electrical nerve stimulation and ultrasound were used to evaluate the location and relationship of the cutaneous nerves to the superior and inferior aspects of the knee in 25 healthy volunteers. Using information derived from these evaluations, investigators defined new linear target treatment areas, or treatment lines, using anatomical landmarks, which were validated against locations of sensory nerves through cadaveric dissection of 15 fresh specimens.

**Results:**

The proposed treatment lines captured the vast majority of nerve branching variations during cadaveric validation.

**Conclusion:**

This study defined treatment lines, identifiable using only anatomical landmarks, which effectively target the nerves responsible for superior and inferior knee pain and reduce the total treatment area and procedure time when administering treatments such as radiofrequency ablation and cryoneurolysis.

## Background

Nerve-targeting treatments for knee pain, such as radiofrequency ablation and percutaneous cryoneurolysis, require treating large areas to ensure coverage of the target nerve(s), due to high variability in the sensory innervation patterns of the knee and limited descriptions of these variations in the literature (Horner & Dellon 1994). Methods of localizing the nerves that contribute to patellar pain include ultrasound imaging and transcutaneous electrical nerve stimulation (TENS). Because the nerves that innervate the patella are normally small (0.5-3 mm in diameter), sonographic visualization can be difficult and time consuming (Gray 2012; Scott 2012). TENS requires searching the entire surface area of the anterior thigh using the TENS wand, which is time consuming as well and may be uncomfortable for the patient. A method of localizing the sensory nerves of the superior and inferior patella using only anatomical landmarks would improve treatment efficiency and tolerability.

Currently, there is no validated anatomical treatment approach that targets the branches of the anterior femoral cutaneous nerve (AFCN) and the lateral femoral cutaneous nerve (LFCN), which innervate the superior aspect of the patella and the surrounding tissue (Scott 2012).

A previous anatomical study in five embalmed cadavers mapped the course of the infrapatellar saphenous nerve (ISN) and medial cutaneous femoral nerve (MFCN), which are responsible for sensory innervation of the inferior aspect of the patella and the surrounding tissue (Le Corroller et al. 2011). Investigators found that the infrapatellar branches of the ISN and the MFCN are located within the area between 55 mm from the medial border of the patella and 44 mm from the medial border of the patellar tendon (Le Corroller et al. 2011). A “treatment box” which targets the branches of ISN and MFCN responsible for inferior patella pain may be defined using these anatomical landmarks as the medial and lateral borders, respectively, and the midline of the patella and top of the tibial tubercle as the superior and inferior borders, respectively. When utilizing this approach to located and treat these nerve branches, the entire length of the treatment box must be treated. This is a large area that includes locations that do not contain nerves of interest, increasing operator time and patient discomfort.

The present study was designed to 1) investigate the locations of the sensory nerves that innervate the superior and inferior patella; 2) using only bony landmarks, define a treatment approach which encompasses all variations of branching patterns of the AFCN, LFCN, MFCN, and ISN as identified using TENS and ultrasound; and 3) validate the accuracy of the treatment approach using cadaveric dissections. This study aims to break new ground by developing the first validated methodology for targeting the nerves that innervate the superior patella. In addition, this study will expand upon previous work targeting the nerves that innervate the inferior patella by defining a more efficient treatment approach.

## Methods

### TENS and ultrasound nerve location study in volunteers

The study protocol was approved by an ethics committee and informed consent was obtained from all individual participants included in the study. All procedures performed in studies involving human participants were in accordance with the ethical standards of the institutional and/or national research committee and with the 1964 Helsinki declaration and its later amendments or comparable ethical standards. Eligible subjects were healthy adults aged 18–60 years with no history of surgery, trauma, or altered anatomy in the measurement areas.

All subjects were examined in the supine position with the knees extended using the SonoSite M-Turbo® Ultrasound System with a 14 MHz linear transducer and the Braun Stimuplex® HNS 12. The ultrasound transducer was placed in the transverse plane with a short axis view of the nerve and held in minimal contact with the skin to ensure that the skin above the nerve was not compressed during measurement. Branches of nerves were identified on the ultrasound as small echogenic honeycomb-shaped structures under the fat layers and on top of the muscle fascia. Nerve branches were located and measured on one or both knees.

#### Innervation of the superior knee

**Fig. 1:**
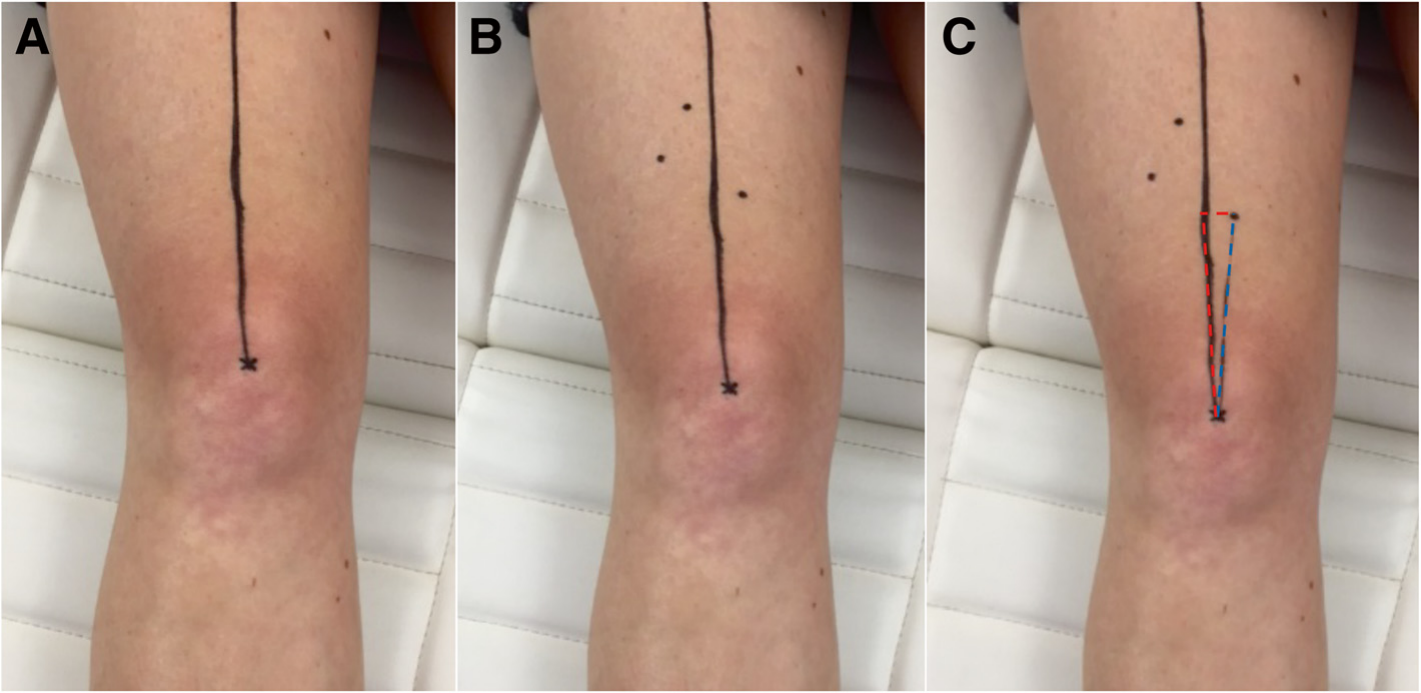
AFCN and LFCN localization and measurement method. A vertical line bisecting the anterior thigh along the sagittal plane is drawn from the center of the patella to the top of the femoral head at the iliac crease (**a**). AFCN and LFCN branches which innervate the superior patella are located using TENS. Black dots on thigh indicate nerve locations (**b**). For each nerve location, the distance to and along the vertical center line is measured, denoted by the red dotted lines. A line is drawn from the nerve location to the center of the knee, denoted by the blue dotted line, and the angle between this line and the vertical center line is measured (**c**)

To locate the AFCN and LFCN, a vertical line bisecting the anterior thigh along the sagittal plane was drawn from the center of the patella to the top of the femoral head at the iliac crease (Fig. 1). The vertical line and the width of the patella were measured. The surface of the thigh was traced with the TENS wand with an initial setting of 1.3 mA, 1.0 ms, and 2.0 Hz. The TENS current was increased in increments of 0.2 mA until the subject reported a sensation in or near the medial portion of the superior edge of the patella. Areas of maximal TENS response for the AFCN branches were located using subject feedback and recorded. These locations were retraced with incrementally lower currents to minimize the radius of stimulation and further specify the nerve location, until pain or sensation in the superior knee was no longer felt by the subject. The final nerve location was marked; this process was repeated for all areas of TENS response for the superior knee. The distance from each nerve location to the vertical center line was measured. A line was drawn from each nerve location to the center of the knee to form an angle with the vertical center line of the thigh, which was measured.

The area between two nerve locations was then traced with the TENS wand in order to discern which nerve locations arose from the same branch. If the subject reported identical sensations when the wand was passed between the nerve locations as when the wand was directly on the nerve locations, it was assumed that the locations were part of the same nerve branch. All nerve locations were confirmed and nerve depths measured using ultrasound. .

#### Innervation of the inferior knee

**Fig. 2:**
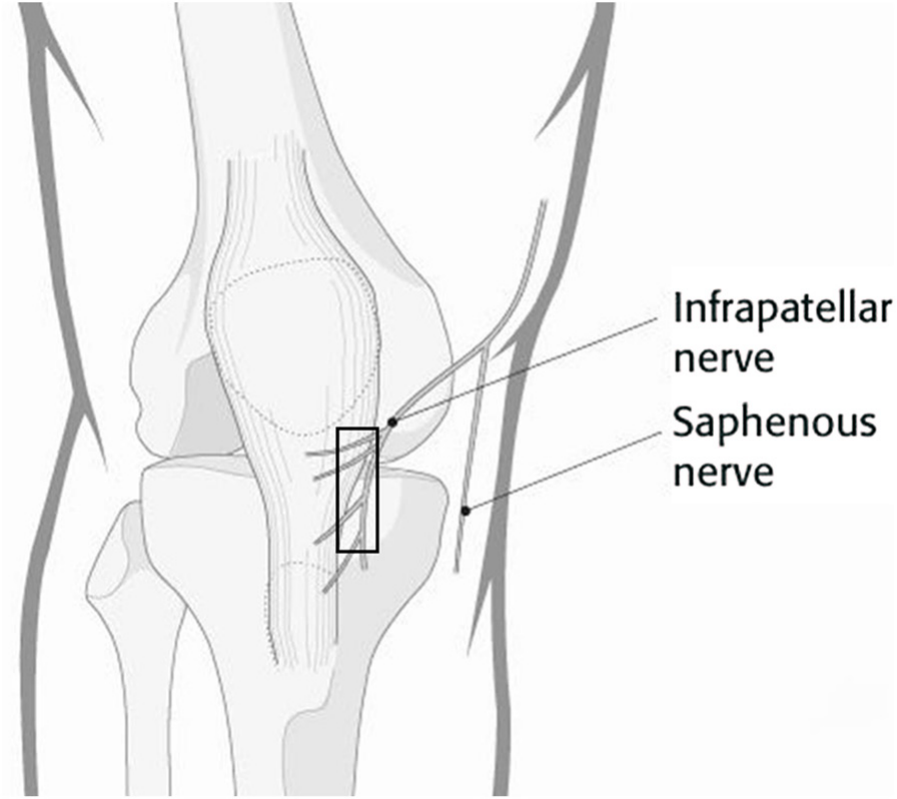
Infrapatellar nerve “treatment box” targeting the ISN and MFCN as defined by Le Corroller. The medial and lateral borders of “treatment box” occur 55 mm medial from the medial border of the patella and 44 mm medial from the medial border of the patellar tendon, respectively. The midline of patella serves as the superior border, and the top of the tibial tubercle the inferior border. Treatment box is not drawn to scale

**Fig. 3:**
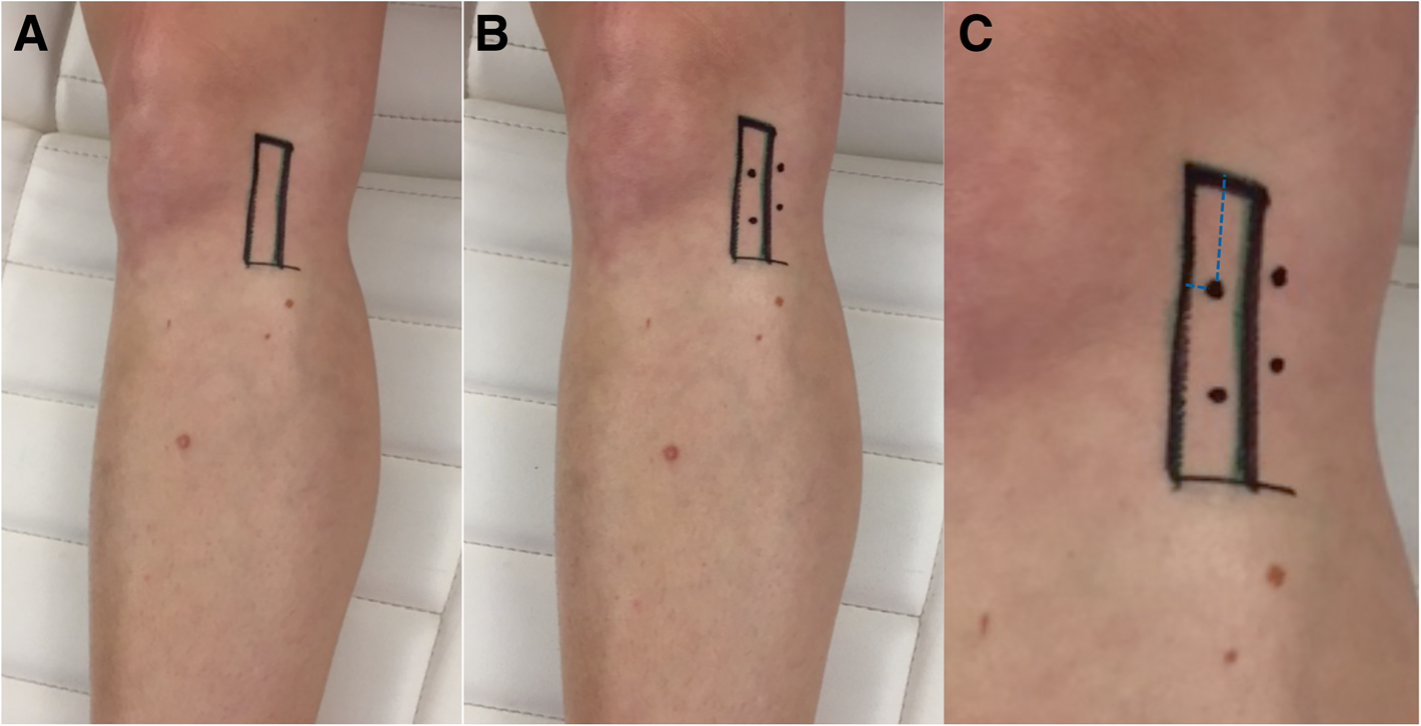
ISN and MFCN localization and measurement method. Le Corroller’s treatment box is drawn (**a**) and nerves are located using TENS within, superior, inferior, and medial to the treatment box. Black dots indicate nerve locations determined using TENS (**b**). The distance from each nerve location to the borders of the treatment box is then measured, indicated by the blue dotted lines (**c**)

To locate the infrapatellar nerve branches innervating the inferior patella, a treatment box (Fig. 2) was drawn on the knee using the anatomical landmarks described by Le Corroller et al., and its dimensions measured (Le Corroller et al. 2011). The surface of the skin within the treatment box was traced with the TENS wand with an initial setting of 1.5 mA, 1.0 ms, and 2.0Hz. Nerve locations were identified using the same technique described for the femoral cutaneous nerves of the superior knee. Each nerve location was marked and its distance from the borders of the treatment box measured. The nerve location and measurement procedure was repeated for all areas of maximal TENS response within the treatment box and at additional infrapatellar nerve locations superior, inferior, and medial to the treatment box (Fig. 3).

After all nerve locations were identified, investigators used the same technique described for the femoral cutaneous nerves to discern which nerve locations arose from the same infrapatellar nerve branch. The infrapatellar nerve location was confirmed and nerve depth measured via ultrasound.

### Cadaveric validation

**Fig. 4:**
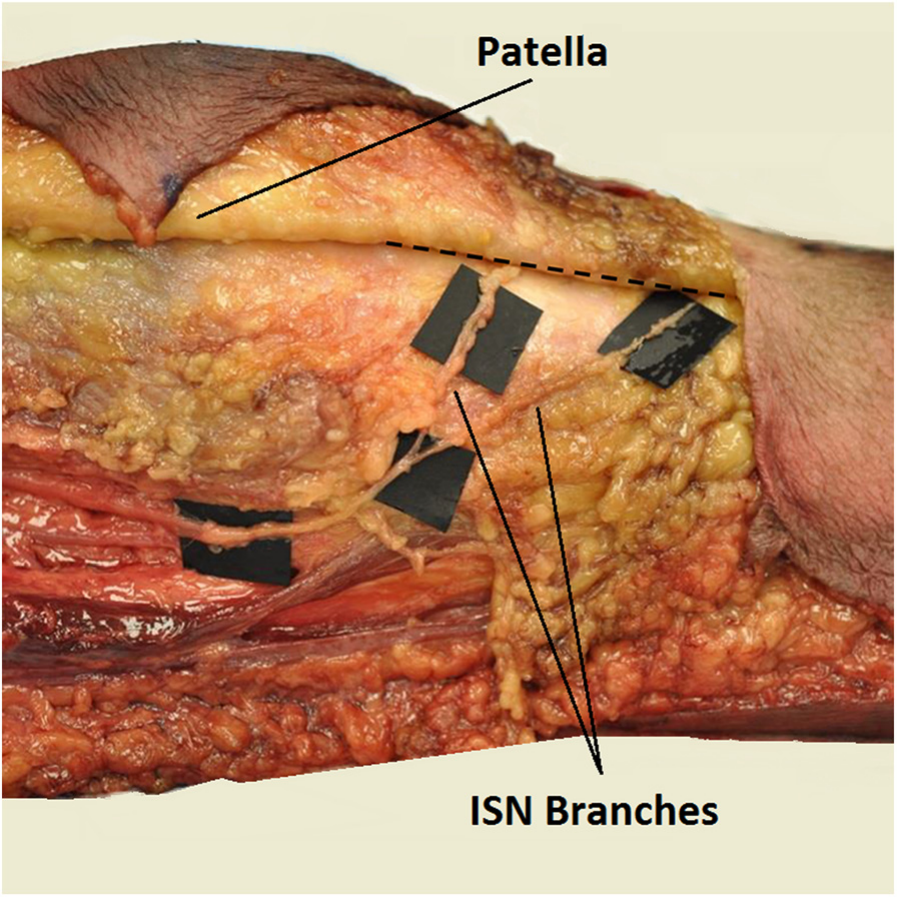
Infrapatellar nerves of the left thigh. Two branches of the ISN cross the treatment line to provide sensory innervation to the inferior knee. Dotted line denotes treatment line

After completion of data analysis from the TENS and ultrasound study, Investigators used the branching patterns that had been identified to propose two linear target treatment areas, or treatment lines, which would optimally target the nerves. These treatment lines were then validated through cadaveric dissection. Prior to dissection, the proposed treatment lines for the nerves innervating the superior and inferior patella were drawn on the skin. All cadavers were inspected to verify the presence of cutaneous nerves at the proposed treatment lines. Dissection began at the anterior superior iliac spine and continued to the region of the superior tibia, inferior to the tibial tubercle. To reveal the presence of the nerves, the skin and fat layers inferior to the treatment line for the femoral cutaneous nerves (superior patella) and medial to the treatment line for the infrapatellar nerves (inferior patella) were removed (Fig. 4).

## Results

For the TENS and ultrasound nerve location study, volunteers were 25 healthy adults (12 females and 13 males) with a mean age of 39 ± 12 years and an average body mass index (BMI) of 27 (6) kg/m^2^.

For the cadaveric validation, fifteen fresh human thigh/knee specimens from 10 cadavers (2 females, 8 males) were dissected. The cadavers were classified into 3 groups by BMI, which was approximated through visual estimation by the orthopedic surgeon performing the dissections. Three specimens were classified as lean (BMI <20 mg/kg^2^), seven as average (BMI 20–30 mg/kg^2^), and five as heavy (BMI >30 mg/kg^2^).

### Innervation of the superior knee

**Fig. 5:**
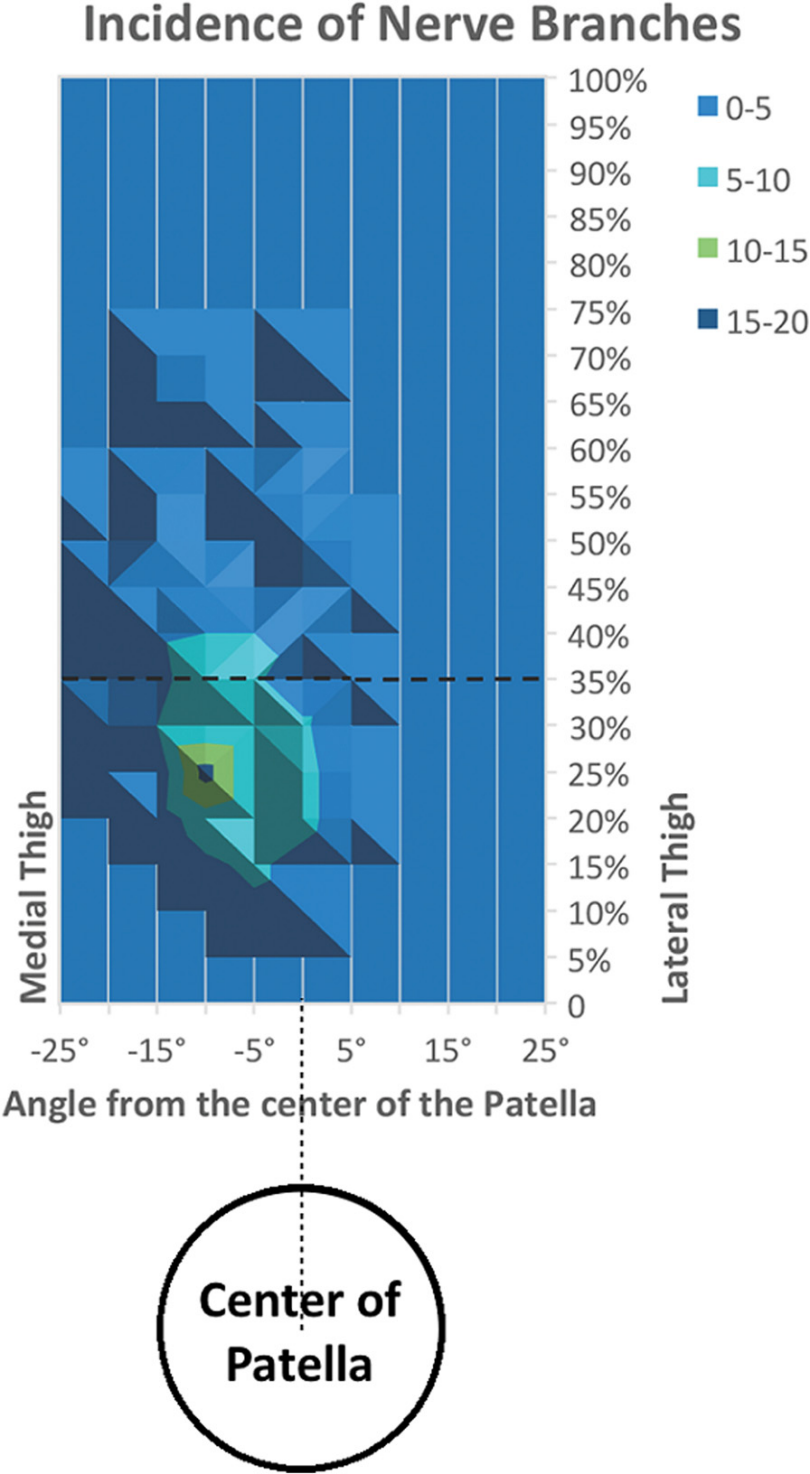
Heat map of the occurrence of the branches of the femoral cutaneous nerve using the left leg where x = 0, y = 0 is the center of the patella. The x-axis represents the angle from the nerve branch to the vertical midline of the patella; the y-axis represents the percent distance of the nerve branch along the thigh; and the z-axis indicates the number of nerve branches occurring at each location. The majority of nerve locations determined using TENS occur at the light blue and green regions of the map. The dotted black line denotes the proposed treatment line

Nerve branches in the superior knee were located and measured in 22 subjects (10 bilateral and 12 unilateral). Using data obtained from TENS and ultrasound nerve localization, investigators created a heat map depicting the population density of nerve branches within the measurement area (Fig. 5). Nerve locations were measured relative to the total length of the vertical center line connecting the center of the patella to the top of the femur. Nerves were identified superior to the center of the patella at a mean distance of 36 % ±13 % of the total length of the vertical center line. The mean distance of the maximum (i.e. furthest from the center of the patella) nerve location for each thigh was on average 45 % ± 13 % along the length of the vertical center line. In 56 % (18/32) of thighs, all nerve locations were found on or medial to the vertical center line and the lines connecting each nerve location to the center of the patella were located between 25° medial and 10° lateral to the center of the knee.

At least one nerve branch was found in 91 % (29/32) and two nerve branches in 50 % (16/32) of thighs. When two branches were identified, they were a mean of 2.7 ± 1.2 cm apart; in all cases, the distance between the branches of the nerves innervating the superior knee was less than the width of the patella at all points along the nerve branches.

**Fig. 6:**
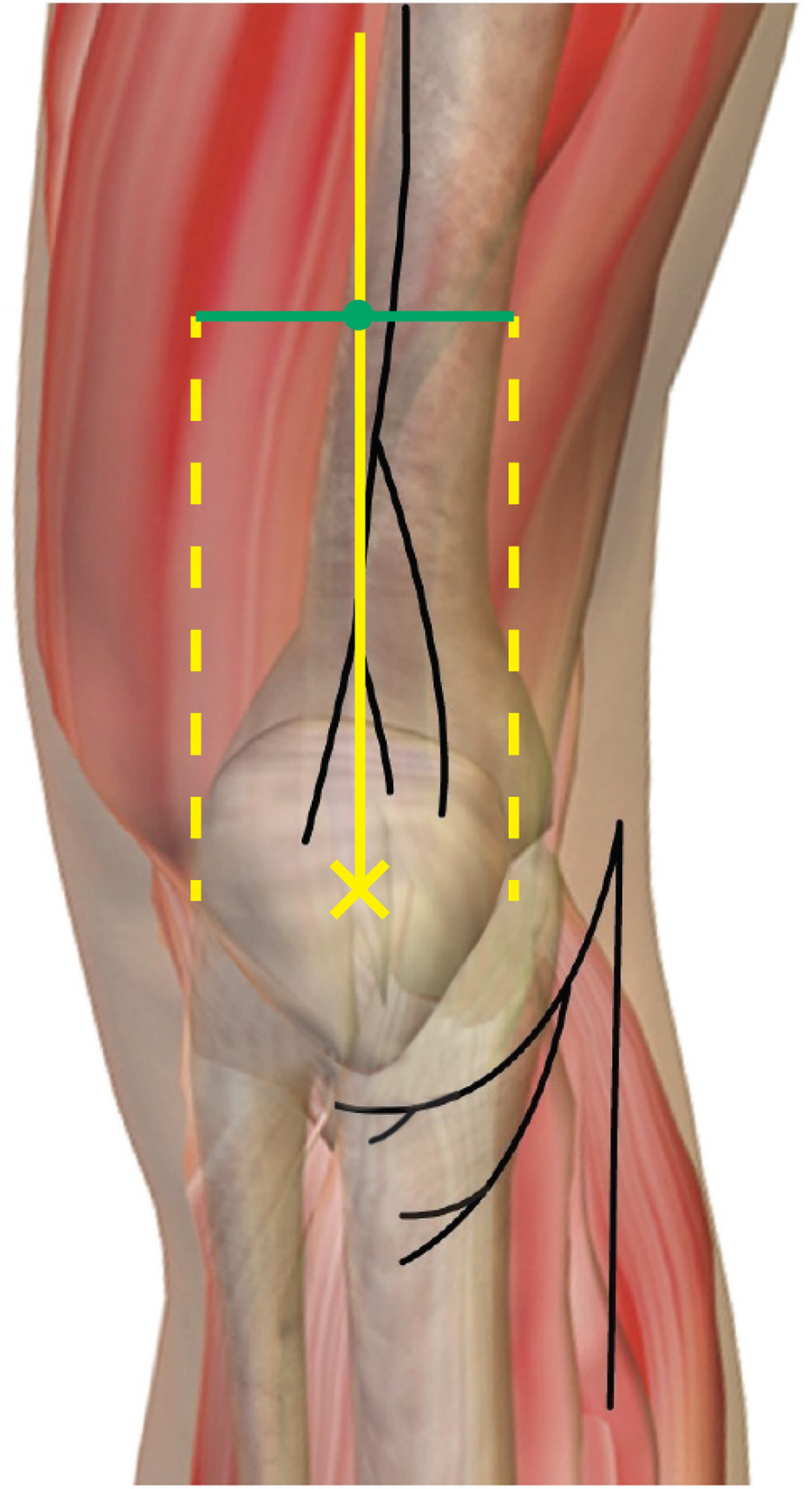
Proposed treatment line for the superior knee. Green line denotes the treatment line. Yellow X denotes the center of the patella. Yellow solid line denotes the vertical midline of the patella. Yellow dotted lines denote the lateral and medial treatment line boundaries, the lateral and medial borders of the patella, respectively. Picture not drawn to scale

The majority (57 %) of nerve locations determined using TENS occurred superior to the center of the patella at a distance of 20–40 % along the length of vertical center line; therefore, it was determined that treatment would be optimally located at a distance of 1/3 along the vertical length of the thigh superior to the knee. At this distance from the center of the patella, the medial and later borders of the patella represent the lines 25° medial and 10° lateral to the center of the knee. Therefore, a proposed horizontal treatment “line” drawn at a distance of 1/3 the vertical length of the thigh superior to the knee and connecting the medial and lateral orders of the patella would encompass the largest occurrence of femoral cutaneous nerves innervating the superior knee (Fig. 6). The proposed horizontal treatment line was validated through cadaveric dissection and was found to intersect the femoral cutaneous nerves in 93 % (14/15) of the cadaver specimens.

### Innervation of the inferior knee

Nerve branches in the inferior knee were located and measured in 24 subjects (19 bilateral and 5 unilateral). Knees were assessed for the number of branches innervating the inferior knee located within the measurement area; there were 14 % (6/43) of knees with one branch, 72 % (31/43) with two branches, 7 % (3/43) with three branches, and 7 % (3/43) for whom no branch patterns could be discerned within the measurement area. Most (83 %; 15/18) subjects had the same number of nerve branches innervating the inferior knee on both lower extremities; there was one subject for whom nerve locations were identified but branching patterns could not be confirmed. Although it is possible that the remaining 17 % of subjects had an asymmetrical number of branches, it is more likely that additional infrapatellar branches of the MFCN or ISN could not be detected by TENS due to their small size. For many subjects, including those with a symmetrical number of nerve branches on both extremities, nerve locations were not necessarily identical on each side. In 37 % (7/19) of assessed bilateral knees, there was a 20 % or greater difference in the distance from the superior border of the treatment box defined using Le Corroller’s methodology for one or more branches of the infrapatellar nerve between the subject’s right and left lower extremity.

**Fig. 7:**
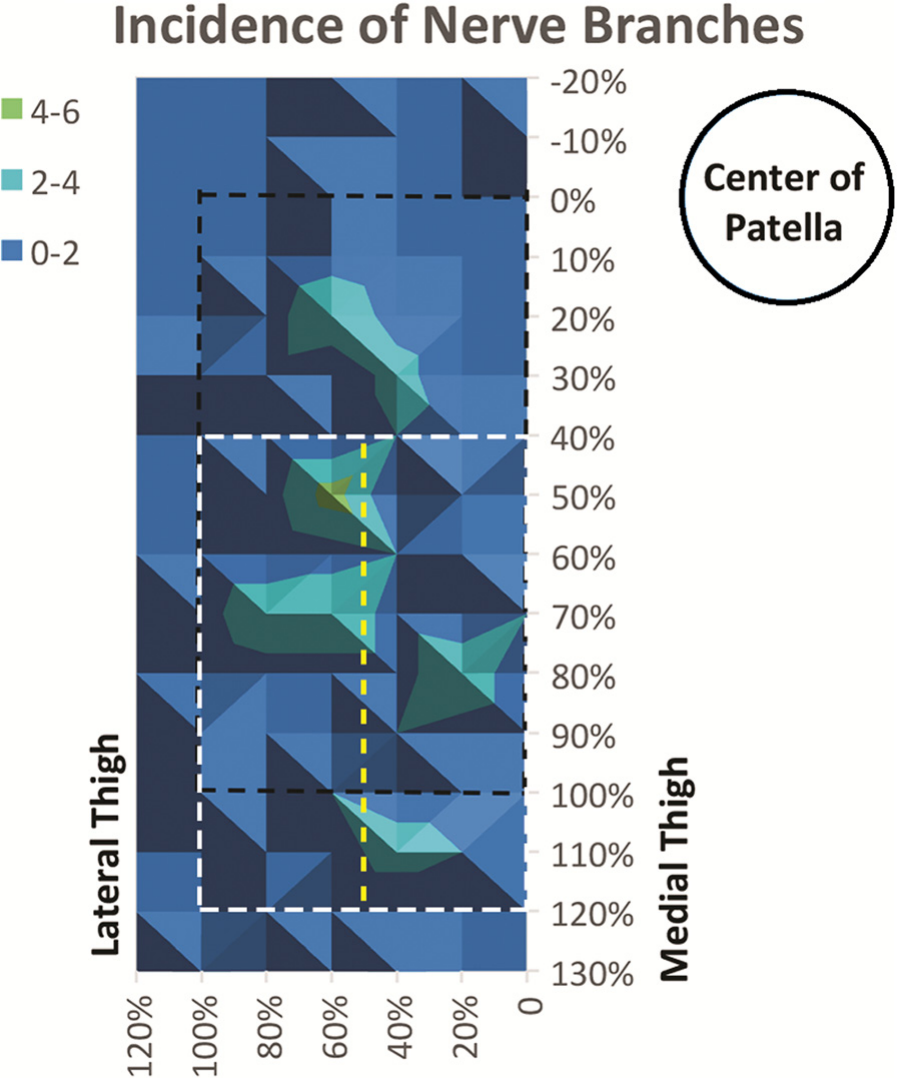
Heat map of the occurrence of the infrapatellar branches of the MFCN and ISN on the right knee. The x-axis represents the percentage of the width of the treatment box lateral to the patella; the y-axis represents the percentage of the length of the treatment box inferior to the patella; and the z-axis indicates the number of nerve branches occurring at each location. The superior, inferior, medial, and lateral boundaries of the treatment box as determined by Le Corroller are defined as y = 0, y = 100, x = 0, and x = 100, respectively, and are denoted by the dotted black box. The majority of nerve locations determined using TENS occur within the area bound by the dotted white box. The dotted yellow line denotes the proposed treatment line

For the 24 subjects (43 knees) measured, the most superior infrapatellar nerve branch was located an average distance of 45 % ± 30 % of the length of the treatment box inferior to the superior border of the treatment box (Fig. 7). The most inferior infrapatellar nerve branch was located an average distance of 15 % ± 25 % of the length of the treatment box superior to the lower border of the treatment box.

**Fig. 8:**
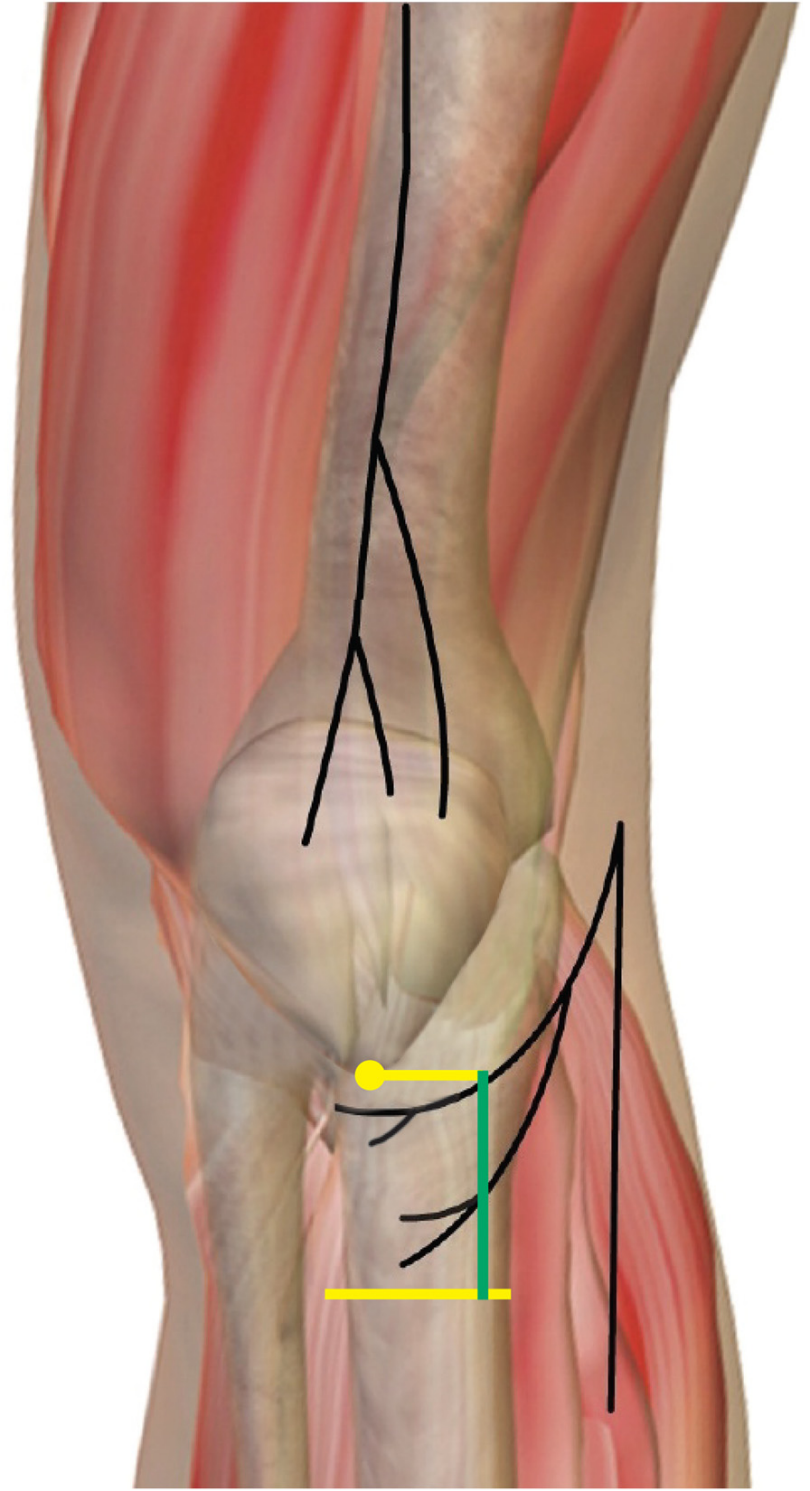
Treatment line for the inferior knee. Picture not drawn to scale. Yellow solid lines denote the superior and inferior borders of the treatment line, the lower pole of the patella and the tibial tubercle, respectively. Green line denotes treatment line, a vertical line which is located 5 cm medial to the lower pole of the patella

Eighty percent of all identifiable infrapatellar nerve branches were located within 40–120 % the length of the treatment box inferior to the upper border (denoted by the white dotted box in Fig. 7). Anatomical landmarks that correlated to these locations were identified as the lower pole of the patella (upper bound) and immediately inferior to the tibial tubercle (lower bound). Thus, a vertical treatment line originating at 50 mm medial to the lower pole of the patella and connecting to the lower pole of the patella at a point just inferior to the tibial tubercle in a straight line would encompass 80 % of the nerve branches innervating the anteromedial aspect of the inferior knee (Fig. 8). The proposed vertical treatment line was validated through cadaveric dissection and was found to intersect the infrapatellar nerves in 93 % (14/15) of the cadaver specimens.

## Discussion

This study is the first to describe a new approach to more efficiently locate and treat the sensory nerves of the superior and inferior knee to relieve pain. Using data derived from TENS and ultrasound localization, investigators defined two treatment lines that can be identified using only anatomical landmarks to target the sensory nerves innervating the superior and inferior knee.

For the nerves of the superior knee, the horizontal treatment line is defined at a distance of 1/3 the vertical length of the thigh superior to the knee and connects the medial and lateral borders of the patella. In cases where the proposed horizontal treatment line does not capture the target nerves, as indicated by an insufficient reduction in superior knee pain, treatment should be extended medially past the medial edge of the patella, as the majority of femoral cutaneous nerve branches occur medial to the vertical centerline of the thigh. The present study was the first to define and validate a treatment line to target the major sensory nerves of the superior patella using anatomical landmarks. This novel approach obviates the need for additional location instrumentation and captures the vast majority of variations in the branching patterns of the targeted sensory nerves in the superior knee.

In addition, this study refined a basic approximation for targeting nerves of the inferior knee developed by Le Correller et al. and validated the new method with a larger sample of cadaver specimens (15 fresh versus 5 embalmed cadavers) than were used by Le Correller et al. (Le Corroller et al. 2011). Results from the TENS location on live volunteers and cadaveric dissections in the present study corroborate findings of previous studies on the innervation and branching patterns in the inferior knee, and confirm that a vertical treatment line at the specified landmarks should allow for the successful treatment of inferior knee pain in most individuals (Kalthur et al. 2015; Kartus et al. 1999; Scott 2012; Tifford et al. 2000). In cases where this line does not capture all targeted branches, suboptimal results for treating inferior knee pain could be improved by extending treatment superiorly along the treatment line to the center of the patella and inferiorly to 1 cm below the tibial tubercle, as a small percentage of nerve branches cross the treatment line at the level of the patella or inferior to the tibial tuberosity. The location of this treatment line is consistent with findings by Kalthur et al., who found that that the infrapatellar nerve branches were located between the lower pole of the patella to under the tibial tubercle in 65.6 % of cases, with the remaining 15.6 % and 18.7 % of cases located near the patella and tibial tuberosity, respectively (Kalthur et al. 2015). In the present study, two branches innervating the inferior knee were located within the measurement area in 72 % of knees, with a single branch in 14 % and three branches in 7 % of knees. These findings are consistent with those of a previous cadaver study, which found that the infrapatellar saphenous nerve had a single branch, two branches, and three branches innervating the inferior patella in 25 %, 62 %, and 10 % of knees, respectively (Kartus et al. 1999).

These treatment lines allow for the treatment of various types of indications, including the treatment of osteoarthritis knee pain in the anterior and medial knee and treatment prior to knee surgery to reduce post-operative pain. In a surgical setting, the sensory innervation of the anterior knee described within this paper covers the workhorse anterior midline knee incision used in surgeries such as total knee replacement. Other potential uses for these treatment lines include treatment of neuropathic pain associated with conditions such as diabetes. However, due to anatomical variability and unpredictability, treating the entirety of the treatment line(s) in all patients may be worthwhile to ensure that all potential branches are targeted. If branches responsible for knee pain remain untreated, clinical results may not be as robust. Further clinical studies are needed to confirm whether treatment of all nerves branches which cross the treatment line is required to provide a meaningful clinical benefit for patients or if treatment of a subset of branches is sufficient.

Limitations of this study include its relatively small sample size of 25 live volunteers and 10 cadaver dissections, with a disproportionately low representation of individuals with BMIs greater than 40 (which compromise the majority of chronic knee pain patients). However, looking at the variation between the existing BMI sub-groups and their local anatomies, we anticipate the anatomy in the higher BMI patients to follow the same pattern as those from < 20 to 40. While we would expect a larger subcutaneous adipose layer, the underlying nerve would follow the same course along the fascia and retinaculum since this is independent of the thickness of the adipose tissue. The limiting factor of treatment within this group may be the technology’s ability to reach deeper nerves rather than expecting a variation in nerve location an anatomy. Further research could include additional cadaver dissections to cover a larger variety of BMIs.

Another limitation of this study was that it excluded subjects with prior knee surgeries, distortions (including scar tissue), or neuropathies in the measurement area as the impact of these conditions on local anatomy variation was unclear. Treating nerves in areas of previous surgery or trauma may be difficult. Clinicians will need to ensure that treatment is proximal to any potential neuroma formation to adequately disrupt transmission of the pain signal, and that the nerve path relative to the treatment line landmarks has not changed as a result of surgery or trauma. Other difficulties may include distorted local anatomy such as bone deformities which could render the treatment line landmarks ineffective. Scar tissue could present multiple difficulties in isolating and distinguishing the targeted nerve using imaging such as ultrasound as well as effectively treating the nerve once found. Further research is needed to assess the impact of deformities, common surgical approaches, and local trauma on nerve location and potential response to treatment.

## Conclusions

This study increases knowledge of the anatomical relationships between the knee region and the cutaneous nerves that innervate it. Despite common variations in the innervation and branching of the femoral, medial, and lateral femoral cutaneous nerves and saphenous nerves, anatomical treatment lines were defined using the results of this study to efficiently and effectively locate and target the sensory nerves implicated in superior and inferior knee pain. The cadaver study confirmed that TENS is a highly accurate method of locating the target nerves on live subjects, as the treatment lines intersected all of the nerves innervating the knee regions most commonly associated with knee pain in 93 % of cadaver specimens. Applying treatment along these treatment lines should effectively target the nerves responsible for superior and inferior knee pain and reduce the total treatment area and procedure time when administering treatments such as radiofrequency ablation and cryoneurolysis.

## Abbreviations

AFCN: Anterior femoral cutaneous nerve
BMI: Body mass index
ISN: Infrapatellar saphenous nerve
LFCN: Lateral femoral cutaneous nerve
MFCN: Medial cutaneous femoral nerve
TENS: Transcutaneous electrical nerve stimulation
